# Exploring barriers to the COVID-19 vaccine in people with psychoses in Western Australia

**DOI:** 10.1016/j.pmedr.2025.103025

**Published:** 2025-03-03

**Authors:** Tammy M. Hall, Katie Attwell, Vera A. Morgan, Susie Hincks, Anna Waterreus

**Affiliations:** a Neuropsychiatric Epidemiology Research Unit, School of Population and Global Health, The University of Western Australia, 35 Stirling Hwy, Perth, WA 6009, Australia.; b VaxPolLab, School of Social Sciences, The University of Western Australia, 35 Stirling Hwy, Perth, WA 6009, Australia.; c Meeting for Minds, 6/25 St. Leonard's St, Mosman, Park WA, 6012, Australia.

**Keywords:** Psychosis, Vaccination, COVID-19, Barriers, Schizophrenia

## Abstract

**Objectives:**

We explored barriers to coronavirus (COVID-19) vaccination among people with psychoses and used these to propose expert recommendations to enhance vaccine uptake in this population.

**Methods:**

A mixed-methods, cross-sectional survey was conducted in Perth, Australia, between September 2022 and September 2023 with 233 adults with psychoses. Participants responded to a short telephone survey comprising questions regarding vaccination status, sources of health information, motivations for vaccine acceptance or refusal, and other factors influencing vaccination decisions.

**Results:**

Most respondents (217, 93.1 %) reported being fully vaccinated, however many described barriers to vaccination that reflected their experiences as people living with mental illness. Over half (126, 54.1 %) said they were not contacted by anyone about getting vaccinated for COVID-19. All participants refusing vaccination denied receiving information on vaccine safety, while almost half of those fully vaccinated had received such information. Unvaccinated participants reported lower levels of trust in general practitioners and psychiatrists compared to those who were vaccinated yet, simultaneously, reported higher levels of trust in other healthcare professionals. Vaccinated individuals cited preventing illness, following recommendations, and compliance with mandates as motivators, while reasons for refusal included vaccine efficacy and side-effect concerns.

**Conclusion:**

Despite limited outreach, participants overcame access barriers, with only those who deliberately sought to refuse the vaccines remaining unvaccinated. Mandates appear to have been a significant contributor to vaccine uptake. Recommendations for encouraging future pandemic and routine vaccinations include upskilling nurses and case managers to provide vaccine information and offering vaccination in mental health facilities and at routine clinical appointments.

## Highlights

• Most respondents were fully vaccinated, despite barriers related to mental illness.
• Over half of participants were not contacted about coronavirus vaccination.
• Unvaccinated participants lacked vaccine safety info, unlike those vaccinated.
• Unvaccinated individuals trusted General Practitioners and psychiatrists less than those vaccinated.
• Mandates were a direct reason for vaccination for one-third of respondents.

## 1 Introduction

The one-month treated prevalence of psychotic disorders in public treatment services in Australia is approximately 3.10 per 1000 people aged 18–64 years (Morgan et al., 2014). Emerging international evidence suggests that individuals with psychoses faced - and continue to face - heightened risks from severe acute respiratory syndrome coronavirus 2 (SARS-CoV-2) infection, including increased rates of hospitalisation and mortality, compared to the general population (Hassan et al., 2022; Mazereel et al., 2021; Vai et al., 2021). This vulnerability is compounded by elevated rates of cardiometabolic disease, premature mortality (De Hert et al., 2009; Saha et al., 2007), and other health conditions prevalent among this population (Foley et al., 2013; Galletly et al., 2012; Morgan et al., 2012). These underlying health disparities likely contribute to worse outcomes from the coronavirus (COVID-19; Földi et al., 2020; Mesas et al., 2020). Despite calls during the height of the pandemic to prioritise this population for COVID-19 vaccination (De Hert et al., 2021; Maguire et al., 2021; Warren et al., 2021), vaccination rates remained low (Bai et al., 2023; Danenberg et al., 2021; Eyllon et al., 2022). In January 2022 in Western Australia, individuals admitted to a mental health unit were six times more likely to be unvaccinated, and seven times more likely to be under-vaccinated than the general population (Hooper et al., 2022). Vaccination rates increased by March 2022, possibly due to the introduction of state-wide restrictions for those not fully vaccinated. However, vaccination rates in inpatient mental health populations remained lower than the general population (Hooper et al., 2022).

An individual's uptake (or not) of available vaccinations can be driven by two broad sets of factors: access and acceptance (Bedford et al., 2018). Relevant access challenges include difficulty scheduling appointments in settings in which an individual feels safe, receiving adequate and appropriate information, and awareness of eligibility (Attwell et al., 2024a). People with psychoses may face additional barriers to assessing this information due to possible cognitive impairments and symptoms of their illness such as delusions and hallucinations. Acceptance factors relate to attitudes about vaccines which are influenced by social networks, prior experiences, perceptions of disease risk, and trust in health authorities (National Centre for Immunisation Research and Surveillance, 2021).

Understanding access and acceptance dynamics is crucial for designing targeted interventions to improve vaccination rates. While both sets of factors can be improved at a systemic level, they require different solutions. By listening to the experiences and perspectives of individuals with psychoses, we can develop effective strategies to enhance vaccine uptake, thereby reducing hesitancy and improving overall health outcomes for this vulnerable population. Accordingly, we offer the first detailed analysis within Australia (to our knowledge) of the extent of, and interplay between, vaccination access and acceptance barriers for people living with psychosis. We then propose expert recommendations to enhance vaccine uptake in this cohort for COVID-19 boosters, other recommended vaccines, and to inform future pandemic planning.

## 2 Methods

### 2.1 Study sample and design

This mixed-methods cross-sectional study was conducted with participants who were first interviewed as part of the 2010 national Survey of High Impact Psychosis (SHIP) Western Australia catchment (Morgan et al., 2012) or the additional 2012 North Metropolitan Perth catchment (Wave 1, Morgan et al., 2013). The Diagnostic Interview for Psychosis (DIP-DM; Castle et al., 2006), a semi-structured clinical interview, was used to classify psychotic illness according to International Classification of Diseases version 10 criteria. Diagnoses were categorised into non-affective psychoses (schizophrenia, delusional disorder, and other non-organic psychoses) and affective psychoses (schizoaffective disorder, bipolar disorder with psychotic features and depressive psychosis). Full participant details are provided elsewhere (Morgan et al., 2021; Morgan et al., 2012). Briefly, Western Australian SHIP participants were part of a Nationally representative sample of adults aged 18–64 years who screened positive for psychosis. The sample included a range of chronicity levels and ages and has been found to be generalisable to adults with psychoses in other developed nations (Morgan et al., 2021).

A short survey was developed, drawing on our expertise in psychotic disorders and vaccination social science, and in association with the Coronavax project, which used qualitative interviews with over 200 participants from various population groups in West Australia to assess attitudes, information, and access needs for the COVID-19 vaccine rollout (Attwell et al., 2021). Building on Coronavax qualitative interview guides and findings (Attwell et al., 2024b; Carlson et al., 2022; Roberts et al., 2023), we worked in conjunction with our research partner with lived experience to design the present survey. The survey comprised open- and closed-ended questions regarding vaccination status, COVID-19 concerns and perceived level of risk, perceived vaccination importance, and other factors influencing vaccination decisions. Follow-up open-ended questions gave respondents the opportunity to provide justification for their responses, with answers transcribed verbatim. Where COVID-19 vaccination reluctance was reported, participants were asked if anything would convince them to accept the vaccine in the future. Experienced mental health researchers contacted participants by telephone between September 2022 and September 2023. Participants verbally provided consent on a voice recorder which was transcribed to a consent form by the interviewer. The survey questions took between five and 10 min to complete. Participants were considered eligible if they were assessed as having the capacity to provide informed consent.

### 2.2 Statistical analyses

Means and standard deviations were computed for continuous variables while categorical variables were expressed in percentages. Group differences were analysed using chi-square tests for categorical variables and independent samples *t*-tests or one-way analysis of variance for continuous variables. All significant alpha levels were set at 0.05 two-tailed. Qualitative data were imported for analysis by the first author. The first and second authors developed an analysis plan augmented by regular meetings to discuss and refine coding strategies and to interpret the results. The number and types of responses were initially coded and analysed for each question, then the entire dataset was analysed inductively to explore recurring themes. Participants could report multiple responses to individual questions and each response was included in analysis for that question. Quantitative analyses were conducted using the Statistical Package for Social Sciences version 28. Qualitative analyses were conducted using NVivo 14.

### 2.3 Ethics statement

Ethics approval was obtained via Human Research Ethics Committees at North Metropolitan Area Mental Health Service (RGS 5416) and the University of Western Australia (2022/ET000551).

## 3 Results

Of the 627 people who participated in SHIP in Western Australia, and gave permission to be recontacted, 77 (12.3 %) had died and 40 (6.4 %) were deemed ineligible. Attempts were made to contact the remaining 510 people and of these 173 (33.9 %) could not be reached after multiple attempts, 104 (20.4 %) declined, and 233 (45.7 %) participated. Two people began the survey but did not complete it; their results were included in analysis where possible. Participants were aged on average 51 years (*Standard Deviation [SD] =* 11.0), 125 (53.6 %) were male and 128 (54.9 %) were diagnosed with schizophrenia. We compared age, sex, and diagnosis for those who completed the survey with those who declined and were unable to be contacted. Although there were no group differences for diagnosis, *χ*^*2*^ (2, *N* = 510) = 2.69, *p* = .26, we observed age, *F* (2, 507) = 9.38, *p* < .001, and sex, *χ*^*2*^ (2, *N* = 510) = 13.57, *p* = .001, differences. Specifically, we were able to contact fewer men than women, with 123 (71.1 %) men unable to be contacted, compared to 125 (53.6 %) who completed the survey and 58 (55.8 %) who declined to participate. We also found that individuals who completed the survey were significantly older (*Mean [M]* = 51.2, *SD* = 11.0) than those who declined to participate (*M* = 46.4, *SD* = 10.3) or could not be contacted (*M* = 47.7, *SD* = 10.3).

Table 1 presents the number of doses of the COVID-19 vaccine participants reported receiving. Those who said they received at least two doses of the vaccine (217, 93.1 %) were considered fully vaccinated, while participants stating they had ceased vaccinating following their first dose (2, 0.9 %) were considered under-vaccinated, and those who had refused the vaccine (14, 6.0 %) were classed as unvaccinated. An individual's vaccination status needed to be contextualised with the timing of our interviews *vis-à-vis* Western Australia's vaccine rollout. At the beginning of data collection, COVID-19 vaccines had been available locally for 18 months, and all participants confirmed that they were aware of their eligibility. None reported being under- or unvaccinated due to access barriers. Therefore, those who were not fully vaccinated at the time of our study had deliberately refused the vaccine or ceased vaccinating. Accordingly, we combined under- and unvaccinated individuals into a single category (refused vaccination) and compared them to those fully vaccinated (accepted vaccination). However, due to the small number of refusers, statistical testing was not undertaken, and we instead report descriptive statistics. While there were no age differences between the groups, there were sex differences. Twelve (75 %) of the refusers identified as female, compared with 96 (44.2 %) of vaccinated individuals. Table 2 presents comparisons between acceptors and refusers, which are further described below and examined according to access and acceptance factors that some participants described facing earlier in the rollout.

**Table 1:** Number of reported coronavirus vaccines received by participants with psychosis in Western Australia, September 2022 – September 2023 (*n* = 233).

Number of reported vaccines	n	%
No vaccines (non-vaccinated)	14	6.0
One vaccine (under-vaccinated)	2	0.9
Two vaccines	31	13.3
Three vaccines	90	38.6
Four vaccines	74	31.8
Five vaccines	22	9.4

n: number of participants, %: percentage of sample.

**Table 2:** Descriptive comparisons of people with psychoses who accepted the coronavirus vaccination with those who refused in Western Australia, September 2022 – September 2023.

	Accepted vaccination *n* = 217		Refused vaccination *n* = 16		Total n = 233	
	n	%	n	%	n	%
Consult and trust about health:						
General Practitioner	145	66.8	9	56.3	154	66.1
Psychiatrist	58	26.7	2	12.5	60	25.8
Other medical or mental health professional	47	22.1	8	50.0	55	23.6
Friends or family	37	17.1	1	6.3	38	16.3
Other (myself, no one, Google)	6	2.8	0	0.0	6	2.6
Contacted about getting vaccinated, *yes*	99	45.6	8	50.0	107	45.9

Received information from General Practitioner, case manager, or doctor, *yes*:						
Vaccine location	70	32.3	5	31.1	75	32.3
Vaccine timing	114	52.5	0	0.0	114	49.1
Vaccine safety, efficacy, side-effects	97	44.7	0	0.0	97	41.8
Worried about contracting COVID-19, *yes*	65	30.0	1	6.3	66	28.3

Perceived risk of contracting COVID-19:						
Very low	42	19.4	4	25.0	46	19.7
Low	85	39.2	6	37.5	91	39.1
Neither low nor high	57	26.3	5	31.3	62	26.6
High	14	6.5	1	6.3	15	6.4
Very high	10	4.6	0	0.0	10	4.3
Unsure	9	4.1	0	0.0	9	3.9

Perceived vaccination importance:						
Low or indifferent perceived importance	30	13.9	11	73.3	41	17.6
Provided qualitative reason	25	11.6	7	46.7	32	13.7
Concerns: vaccine safety or side-effects	4	1.9	2	13.3	6	2.6
Concerns: vaccine efficacy	5	2.3	1	6.7	6	2.6
Received vaccine due to mandates	5	2.3	0	0	5	2.1
High perceived importance	181	83.8	1	6.7	182	78.1
Provided qualitative reason	181	83.8	1	6.7	182	78.1
Prevent illness in self and others	119	55.1	0	0	119	51.1
Provides peace of mind	28	13.0	0	0	28	12.0
Mandatory requirements or restrictions	27	12.5	0	0	27	11.6
Concerns: vaccine safety or side-effects	2	0.9	1	6.7	3	1.3

n: number of participants, %: percentage of sample, COVID-19: coronavirus.

### 3.1 Access barriers and strategies to address

While no participant reported access barriers as a current reason for lack of vaccination, many did report barriers obtaining the vaccine. Over half of all respondents (126, 54.1 %) said they had not been contacted by anyone regarding getting vaccinated for COVID-19. Acceptors and refusers alike said they received similar information from their case manager or doctor about which sites they could attend to receive the vaccines. However, all refusers denied receiving information on vaccine timing, safety, effectiveness, or side-effects. By contrast, close to half of the acceptors reported receiving information on timing and safety (52.5 % and 44.7 %, respectively). Despite this, most participants (194, 83.6 %), including most of the refusers (13, 81.3 %), said they felt they received sufficient information about COVID-19 vaccines.

Respondents were given the opportunity to provide suggestions on information they would have liked to receive, and on how to make future vaccinations easier to access. Ninety people (38.6 %) provided such suggestions. Of these, 38 (42.3 %) recommended accessibility improvements, such as more pharmacists and general practitioners administering the vaccine; more vaccination centres; home or workplace visits for vaccine administration; vaccination clinics specifically for mental health patients; and alternatives to online booking systems. In addition, 26 (28.9 %) suggested more effective communication and promotion regarding the vaccine, while 22 (24.4 %) sought more product-related information such as information about side-effects and the differences between vaccine brands.

### 3.2 Acceptance barriers and motivators to vaccinate

Trust emerged as an important issue distinguishing vaccine refusers from acceptors. Slightly fewer refusers reported trusting their general practitioner or psychiatrist regarding their health compared to those who accepted. However, at the same time, refusers reported *higher* levels of trust in other medical or mental health professionals, including their carer or case worker, compared to acceptors.

Among those who were vaccinated, motivations included: a desire to prevent illness in self and others (98, 45.2 %); recommendations from healthcare professionals, the media, or friends and family (88, 40.6 %); compliance with mandates (67, 30.9 %); and the easy accessibility of vaccines (10, 4.6 %). Mandates were cited as a direct reason for vaccination by 69 people (31.5 %), including the two refusers who had initially accepted one vaccine dose. Mandates were the sole reason for vaccination by 54 participants (24.7 %). Of those who cited mandates as a reason for vaccination, 26 (37.7 %) discussed work requirements, 13 (18.8 %) sought to access public spaces, and nine(13.0 %) identified travel or the desire for borders to open. Three acceptors and the two under-vaccinated individuals (2.3 %), said they felt forced to have the COVID-19 vaccine due to the mandates.

Reasons for vaccine refusal included: concern regarding vaccine side-effects (10, 62.5 %); lack of desire to take the vaccine (4, 25 %); religious objections (1, 6.3 %); and medical advice (1, 6.3 %).

Table 2 presents participants' reported reasons for why they saw the vaccine as important or not, broken down into acceptors and refusers. Some people who refused the vaccine nevertheless perceived the disease as serious or saw vaccination as important. One unvaccinated individual reported concern over contracting COVID-19 and perceived themselves at high risk for catching the illness but had nonetheless chosen not to take the vaccine. This person declined to answer questions regarding factors influencing their vaccination decisions, but stated they would have liked information on alternative treatment options. Another unvaccinated participant reported believing that vaccination was very important, but stated they were advised not to get vaccinated by a family member due to concerns regarding the safety of the COVID-19 vaccine.

Among our participants, both acceptors (48, 22.1 %) and refusers (13, 81.3 %) talked about their worries and fears about the vaccines. Over half of these (35, 57.4 %) focused on potential side effects, while a smaller number (7, 11.5 %) expressed concerns about the vaccines not being effective. Ten acceptors (20.8 %) stated that these fears resulted in them intentionally delaying getting the vaccine. An additional 33 people (41.2 %) reported concerns around scaremongering, misinformation, and mixed messaging.

Regarding intent to receive further COVID-19 vaccinations, 74 (34.1 %) people reported being either up to date or intending to receive their next vaccine, 63 (29.0 %) did not intend to have any more vaccinations, and a further 27 (12.4 %) were undecided. Only two (14.3 %) of the 14 unvaccinated individuals indicated a willingness to be vaccinated against COVID-19 in the future. One said they would be willing following longer-term data collection, and the other cited a lifestyle change resulting in reduced isolation and more exposure to people.

## 4 Discussion

Due to previous findings in the literature, we expected people with psychoses to be less likely to have been vaccinated with the COVID-19 vaccine than the general population (Aitken et al., 2023; Hooper et al., 2022; Maguire et al., 2021). However, our findings did not support this. Despite half of our participants not being directly approached to take a vaccine, by the time we spoke to them, all who were willing to be vaccinated had managed to overcome any access barriers and complete at least a two-dose course. Indeed, most had received the three doses of vaccine recommended at the time by authorities and included in the state's employment vaccine mandates. One explanation for this could be that the timing of our study, where vaccines had been available for 18 months, provided sufficient time for participants to overcome access barriers and obtain vaccination if they desired to do so. It is also possible that increased pressure from the Government and media to obtain the COVID-19 vaccine compared to other vaccines may have contributed to increased vaccination rates. Further, differing vaccination rates have been found in varying countries with different healthcare systems (Peritogiannis et al., 2023; Tzur Bitan et al., 2021), as well as among inpatients compared to outpatients (Bai et al., 2023; Danenberg et al., 2021). It is also possible that Western Australia's vaccine mandates, requiring staff in many professions to be fully vaccinated, as well as restrictions placed on entering public spaces for those not fully vaccinated, may be at least partly responsible for our high levels of vaccination compared to previous studies. Mandates have been found to be a driver of uptake for vaccines in other settings (Attwell et al., 2020; Lévy-Bruhl et al., 2019; Vaz et al., 2020), as well as for COVID-19 vaccines (Fitzpatrick et al., 2023; Mills and Rüttenauer, 2022; Roberts et al., 2023). Almost a third of our participants identified West Australia's mandates among their motivations for being vaccinated, with mandates being the sole reason given by one quarter of them. Scholars and frontline vaccination staff often debate whether mandates are reasonable and proportional policy instruments, especially because they can produce psychological reactance (a motivational drive to restore perceived loss of freedom, Rosenberg and Siegel, 2018) and can contribute to social and political polarisation (Bardosh et al., 2022). Consequently, many prefer voluntarism underpinned by strong state programs that can deliver effective vaccination outreach. Our participants did not receive this kind of outreach, which would have been optimal. Given this, our findings indicate that mandates may be important to attaining vaccination among cohorts who face barriers to vaccination, and who may otherwise be at risk from infection, severe illness, and death. Mandates can have communicative power, indicating that governments believe vaccination is highly important. They can also help to move vaccination higher on the “to do” list or motivate people to seek out vaccination even when the health and life challenges they face might otherwise impede this. Indeed, very few of those who accepted vaccination reported feeling pressured to do so. This finding may indicate that, for this cohort, mandates do more good than harm in an emergency setting, where programs struggle to reach all members of the population with an offer of vaccination quickly.

However, using other interventions is also important, especially outside of an emergency setting where vaccination policies tend to be more voluntaristic. To improve uptake in this population, governments should seek to upskill nurses, case managers, and support workers to provide appropriate advice regarding vaccination; for some professionals this will include helping them to answer questions about side-effects, which may help allay fears regarding vaccine safety and effectiveness. This is particularly important as concern regarding side-effects was the main reason for refusal or delayed vaccination among our participants. It was also the main reason for participants not intending to take vaccines in the future, consistent with previous findings (Walton et al., 2022). Our participants suggested that governments should open vaccination sites in hospitals or at mental health services, run by staff experienced in working with people with severe mental illness, as a way of reducing access barriers. This has been found to be effective in other countries (Miles et al., 2020). Offering vaccinations while patients are attending routine clinical appointments could further help to reduce access barriers in this population (Tzur Bitan et al., 2021).

### 4.1 Limitations

While the original cohort of SHIP participants has been found to be representative of adults living with psychosis in developed nations (Morgan et al., 2021), our interviewed sample was found to include more females and be older than those who did not participate. It is possible that males and younger people may have different views to those expressed here. Further, our participants were primarily from Western Australia, which was in a unique position during the pandemic. The state experienced a hard lockdown and therefore had few cases of COVID-19 in the early days of the pandemic. This may have led participants to perceive the risk of infection as low, possibly contributing to delayed vaccination. Further, as our findings do not support previous research suggesting people with psychoses are less likely to be vaccinated than the general population (Bai et al., 2023; Danenberg et al., 2021; Eyllon et al., 2022), it is unclear how generalisable our results are to other parts of Australia or the world. Finally, vaccination status was self-reported and not verified by medical records. It is possible that participants may have misremembered the number of vaccines they had received so, for some participants, the information recorded may not have been accurate.

### 4.2 Conclusion

Despite these limitations, our study offers a comprehensive account of the views of Australians living with psychoses regarding access and acceptance barriers to the COVID-19 vaccine. We have highlighted the importance of including the views of these populations in service planning, and have offered suggestions for improving vaccination rates, for COVID-19 boosters, as well as for vaccines of other communicable diseases, and for future pandemic planning.

## Funding sources

This research was supported by the Western Australian Future Health Research and Innovation Fund, which is an initiative of the Western Australian State Government. This source was not involved in study design; collection, analysis, and interpretation of data; the writing of the report; or the decision to submit for publication.

## CRediT authorship contribution statement

**Tammy M. Hall:** Writing – review & editing, Writing – original draft, Visualization, Investigation, Formal analysis, Data curation. **Katie Attwell:** Writing – review & editing, Methodology, Formal analysis. **Vera A. Morgan:** Writing – review & editing, Methodology. **Susie Hincks:** Writing – review & editing, Methodology. **Anna Waterreus:** Writing – review & editing, Supervision, Software, Project administration, Methodology, Investigation, Funding acquisition, Conceptualization.

## Declaration of competing interest

The authors declare that they have no known competing financial interests or personal relationships that could have appeared to influence the work reported in this paper.

## Data Availability

The data underlying this article will be shared on reasonable request to the corresponding author.
